# Supplementary material for: PRRT2 as an auxiliary regulator of Nav channel slow inactivation
Source: eLife. 2026 Sep 11;14:RP109327. doi: 10.7554/eLife.109327 (PMC13568701; doi:10.7554/eLife.109327)
Supplement: Supplementary file 3 [file elife-109327-fig1-data2.zip › Figure 1-Source data 2.pdf]

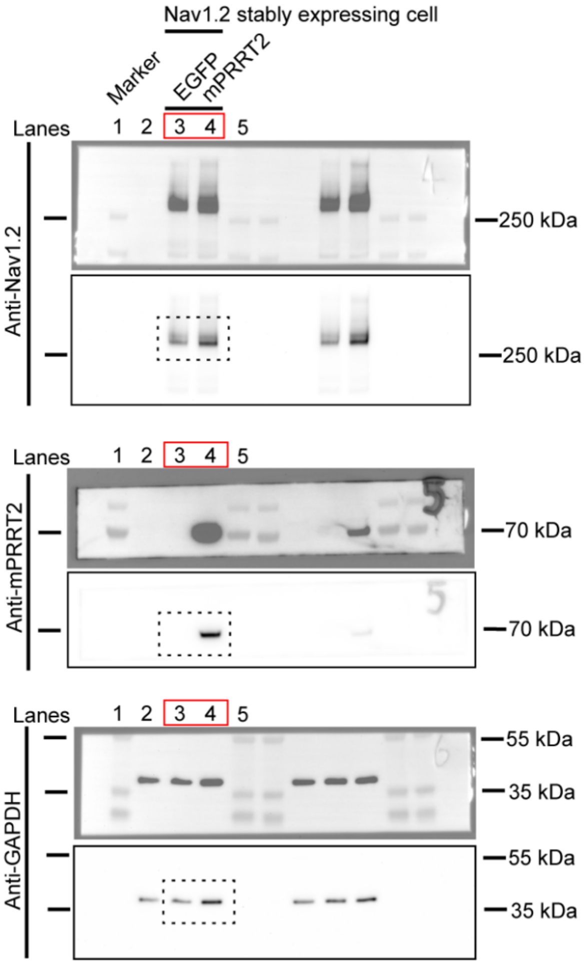

Lanes :1, marker; 2, HEK293T+EGFP; 3, Nav1.2 stably expressing cell +EGPF; 4, Nav1.2 stably expressing cell + mPRRT2; 5, marker.
