# Supplementary material for: PRRT2 as an auxiliary regulator of Nav channel slow inactivation
Source: eLife. 2026 Sep 11;14:RP109327. doi: 10.7554/eLife.109327 (PMC13568701; doi:10.7554/eLife.109327)
Supplement: Supplementary file 5 [file elife-109327-fig2-figsupp1-data2.zip › Figure 2-figure supplement 1-Source Data 2.pdf]

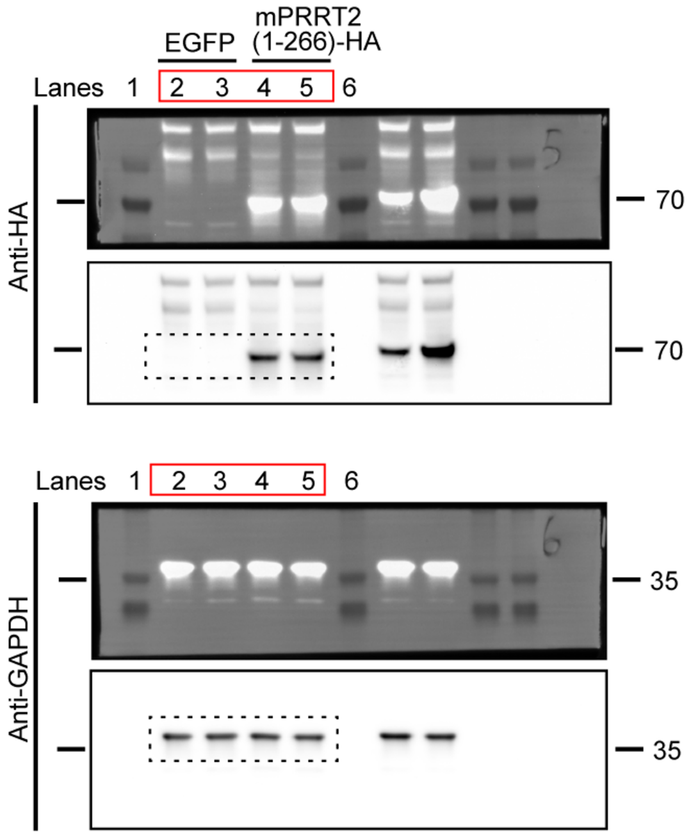

Lanes :1, 6: marker
2, 3: EGPF
4, 5: mPRRT2 (1-266)-HA
