# Supplementary material for: PRRT2 as an auxiliary regulator of Nav channel slow inactivation
Source: eLife. 2026 Sep 11;14:RP109327. doi: 10.7554/eLife.109327 (PMC13568701; doi:10.7554/eLife.109327)
Supplement: Supplementary file 11 [file elife-109327-fig5-data14.zip › Figure 5-source data 14.pdf]

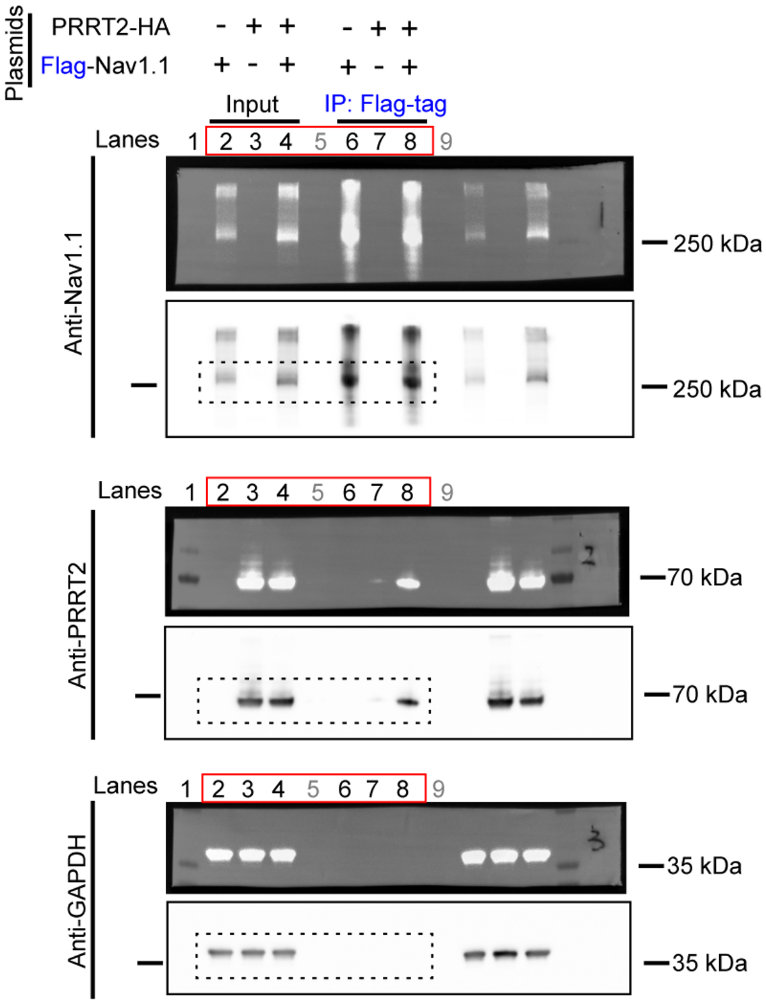

Lanes :1, marker; 2-4, Input; 6-8, IP Flag-tag; 5 and 9, blank.
