# Supplementary material for: PRRT2 as an auxiliary regulator of Nav channel slow inactivation
Source: eLife. 2026 Sep 11;14:RP109327. doi: 10.7554/eLife.109327 (PMC13568701; doi:10.7554/eLife.109327)
Supplement: Supplementary file 23 [file elife-109327-fig6-data2.zip › Figure 6-source data 2.pdf]

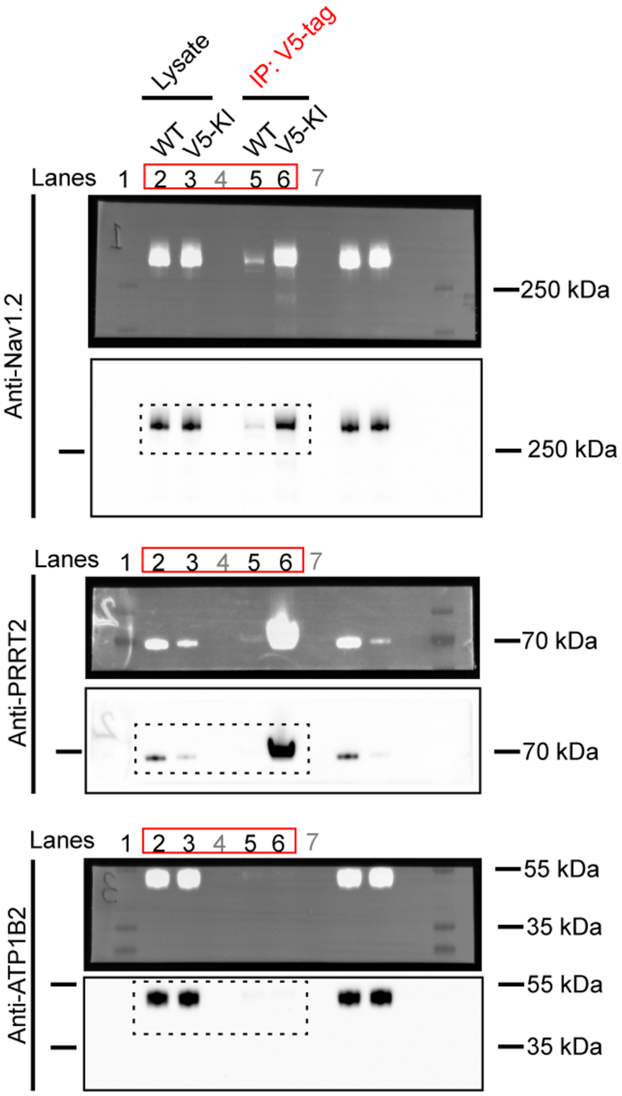

Lanes :1, marker; 2 and 3, Lysate; 5 and 6, IP V5-tag; 4 and 7, blank.
2, 5: wild-type (WT)
3, 6: *Prrt2-V5* knock-in (V5-KI)
