# Supplementary material for: PRRT2 as an auxiliary regulator of Nav channel slow inactivation
Source: eLife. 2026 Sep 11;14:RP109327. doi: 10.7554/eLife.109327 (PMC13568701; doi:10.7554/eLife.109327)
Supplement: Peer review history

# Editor report: eLife Assessment

**DOI:** 10.7554/eLife.109327.4.sa0

**Authors:** Kenton J Swartz

**Affiliations:** National Institute of Neurological Disorders and Stroke United States

**Keywords:** Compelling, Important

This **important** study identifies PRRT2 as an auxiliary regulator of Nav channel slow inactivation in vitro and in vivo, demonstrating that PRRT2 facilitates entry into, and delays recovery from, the slow-inactivated state. The revised manuscript has been substantially strengthened, providing **compelling** evidence that PRRT2 is relevant to normal brain physiology and disease pathophysiology, providing a mechanistic link between PRRT2 mutations and episodic neurological phenotypes. Overall, this study will be of interest to ion channel biophysicists and neurophysiologists, particularly those studying channelopathies.

# Referee report: Reviewer #1 (Public review)

**DOI:** 10.7554/eLife.109327.4.sa1

[Editors' note: this version has been assessed by the Reviewing and Senior Editor without further input from the original reviewers. The authors have addressed the comments raised in the previous round of review.]

Summary:

The manuscript by Lu and colleagues demonstrates convincingly that PRRT2 interacts with brain voltage-gated sodium channels to enhance slow inactivation in vitro and in vivo. The work is interesting and rigorously conducted. The relevance to normal physiology and disease pathophysiology (e.g., PRRT2-related genetic neurodevelopmental disorders) seems high. Some simple additional experiments could elevate the impact and make the study more complete.

Strengths:

Experiments are conducted rigorously including experimenter blinding and appropriate controls. Data presentation is excellent and logical. The paper is well written for a general scientific audience.

Comments on revised version.

The manuscript by Lu and colleagues has been revised sufficiently to address all my prior concerns.

Experiments are conducted rigorously including experimenter blinding and appropriate controls. Data presentation is excellent and logical. The paper is well written for a general scientific audience.

# Referee report: Reviewer #2 (Public review)

**DOI:** 10.7554/eLife.109327.4.sa2

Summary:

As a member of DspB subfamily, PRRT2 is predominantly expressed in CNS and has been associated with various paroxysmal neurological disorders. Previous studies have shown that PRRT2 interacts with Nav and Cav channels, modulating channel properties and neuronal excitability.

In this manuscript, Lu et al. demonstrate that PRRT2 is a potent regulator of Nav channel slow inactivation, promoting the development of Nav slow inactivation and impeding the recovery from slow inactivation. This effect is highly conserved in PRRT2s across species as well as among DspB family members (TRARG1 and TMEM233). The authors further confirmed the interaction between Nav channels and PRRT2 in heterologous expression systems as well as in Prrt2-V5 knock-in mice. Prrt2-mutant mice, which lack PRRT2 expression, require lower stimulation thresholds for evoking after-discharges when compared with WT mice.

Overall, this is a well-executed and methodologically comprehensive study. This work offers valuable insight into the physiological functions of PRRT2 and reveals a potential pathogenic mechanism underlying PRRT2-associated neurological disorders.

The revised manuscript has addressed most of the concerns raised by the reviewers and has been substantially strengthened, although I still have several concerns regarding the discussion section.

Strengths:

(1) Overall, this is a well-executed and methodologically comprehensive study. The electrophysiological data strongly support the conclusion that PRRT2 is a potent regulator of Nav channel slow inactivation. The observation that this regulation is conserved in PRRT2 across species and among DspB family members raises the possibility that altered regulation of Nav channels may also contribute to the pathogenesis of TRARG1- or TMEM233-associated disorders.

(2) Co-immunoprecipitation assay performed using brain tissue from genetically modified Prrt2-V5 knock-in mice provides convincing in vivo evidence for the interaction between PRRT2 and Nav1.2 channels.

(3) Prrt2-V5 KI mice show markedly reduced PRRT2 protein expression and display phenotypes similar to those observed in Prrt2-mutant mice, supporting an important role of PRRT2 in regulating neuronal and network excitability.

Weaknesses:

(1) Nav1.6 is also highly expressed in cortical neurons and is widely regarded as a major contributor to action potential initiation and sustained high-frequency firing. Given that PRRT2 similarly regulates the fast and slow inactivation of Nav1.6 and Nav1.2 channels, the potential contribution of Nav1.6 regulation to neuronal and network excitability should be discussed.

(2) Slow inactivation is generally considered to develop over timescales ranging from hundreds of milliseconds to seconds or longer. Therefore, the statement in Discussion (Page 13, line 381-382) that "slow inactivation develops on a timescale of tens of milliseconds to seconds" may not accurately reflect the conventional kinetic definition of slow inactivation and should be clarified.

(3) Page 14, line 417-430: "question about how Nav channel slow inactivation is regulated in cells that do not express PRRT2".

PRRT2 is unlikely to be the sole regulator of Nav channel slow inactivation. Other molecules and signaling pathways may regulate Nav channel and contribute to neuronal excitability. In addition, neuronal excitability can also be regulated through modulating other Nav properties, such as long-term inactivation or slow recovery from inactivation, as well as through modulating the activity of other ion channels, for example, Kv7.2 and Kv7.3 channels. Therefore, PRRT2-negative cells may utilize alternative mechanisms to fine-tune neuronal excitability. In its current form, this paragraph somewhat overstates the role of PRRT2 and would benefit from a more balanced discussion.

(4) Page 50, Figure 7-figure supplement 2: It would be helpful to include representative traces of the 1st and the last (20th) compound APs in panels B and C.

# Referee report: Reviewer #3 (Public review)

**DOI:** 10.7554/eLife.109327.4.sa3

This paper reveals that the neuronal protein PRRT2, previously known for its association with paroxysmal dyskinesia and infantile seizures, modulates the slow inactivation of voltage-gated sodium ion (Nav) channels, a gating process that limits excitability during prolonged activity. Using electrophysiology, molecular biology, and mouse models, the authors show that PRRT2 accelerates entry of Nav channels into the slow-inactivated state and slows their recovery, effectively dampening excessive excitability. The effect seems evolutionarily conserved, requires the C-terminal region of PRRT2, and is recapitulated in cortical neurons, where PRRT2 deficiency leads to hyper-responsiveness and reduced cortical resilience in vivo. These findings extend the functional repertoire of PRRT2, identifying it as a physiological brake on neuronal excitability. The work provides a mechanistic link between PRRT2 mutations and episodic neurological phenotypes.

Comments:

(1) The precise structural interface and the molecular basis of gating modulation remain inferred rather than demonstrated.

(2) The in vivo phenotype reflects a complex circuit outcome and does not isolate slow-inactivation defects per se.

(3) Expression of PRRT2 in muscle or heart is low, so the cross-isoform claims are likely of limited physiological significance.

(4) The mechanistic separation between trafficking of PRRT2 and its gating effects is not clearly resolved.

(5) Additional studies with Nav1.6 should be carried out.

Comments on revised version.

These comments have been addressed in the revised version.

# Author comment: Author response

**DOI:** 10.7554/eLife.109327.4.sa4

**Authors:** Bin Lu, Qi-Wu Xu, Jing Zhang, Xue-Mei Wu, Jun-Yan He, Jing-Qiu Peng, Guang Yang, Ke-Xian Li, Ling Zhuang, Yu-Xian Zhang, Zhi-Ying Wu, Zhi-Qi Xiong

**Affiliations:** Center for Excellence in Brain Science and Intelligence Technology Shanghai China; Center for Excellence in Brain Science and Intelligence Technology Shanghai China; Center for Excellence in Brain Science and Intelligence Technology Shanghai China; Center for Excellence in Brain Science and Intelligence Technology Shanghai China; Center for Excellence in Brain Science and Intelligence Technology Shanghai China; Center for Excellence in Brain Science and Intelligence Technology Shanghai China; Center for Excellence in Brain Science and Intelligence Technology Shanghai China; Fudan University Shanghai China; Center for Excellence in Brain Science and Intelligence Technology Shanghai China; Center for Excellence in Brain Science and Intelligence Technology Shanghai China; Zhejiang University School of Medicine Hangzhou China; Center for Excellence in Brain Science and Intelligence Technology Shanghai China

The following is the authors’ response to the original reviews.

**Reviewer #2 (Public review):**

Summary:

As a member of DspB subfamily, PRRT2 is predominantly expressed in CNS and has been associated with various paroxysmal neurological disorders. Previous studies have shown that PRRT2 interacts with Nav and Cav channels, modulating channel properties and neuronal excitability.

In this manuscript, Lu et al. demonstrate that PRRT2 is a potent regulator of Nav channel slow inactivation, promoting the development of Nav slow inactivation and impeding the recovery from slow inactivation. This effect is highly conserved in PRRT2s across species as well as among DspB family members (TRARG1 and TMEM233). The authors further confirmed the interaction between Nav channels and PRRT2 in heterologous expression systems as well as in Prrt2-V5 knock-in mice. Prrt2-mutant mice, which lack PRRT2 expression, require lower stimulation thresholds for evoking after-discharges when compared with WT mice.

Overall, this is a well-executed and methodologically comprehensive study. This work offers valuable insight into the physiological functions of PRRT2 and reveals a potential pathogenic mechanism underlying PRRT2-associated neurological disorders.

The revised manuscript has addressed most of the concerns raised by the reviewers and has been substantially strengthened, although I still have several concerns regarding the discussion section.

Strengths:

(1) Overall, this is a well-executed and methodologically comprehensive study. The electrophysiological data strongly support the conclusion that PRRT2 is a potent regulator of Nav channel slow inactivation. The observation that this regulation is conserved in PRRT2 across species and among DspB family members raises the possibility that altered regulation of Nav channels may also contribute to the pathogenesis of TRARG1- or TMEM233-associated disorders.

(2) Co-immunoprecipitation assay performed using brain tissue from genetically modified Prrt2-V5 knock-in mice provides convincing in vivo evidence for the interaction between PRRT2 and Nav1.2 channels.

(3) Prrt2-V5 KI mice show markedly reduced PRRT2 protein expression and display phenotypes similar to those observed in Prrt2-mutant mice, supporting an important role of PRRT2 in regulating neuronal and network excitability.

We sincerely thank the reviewer for the meticulous evaluation of our revised manuscript and for the constructive and insightful comments.

Weaknesses:

(1) Nav1.6 is also highly expressed in cortical neurons and is widely regarded as a major contributor to action potential initiation and sustained high-frequency firing. Given that PRRT2 similarly regulates the fast and slow inactivation of Nav1.6 and Nav1.2 channels, the potential contribution of Nav1.6 regulation to neuronal and network excitability should be discussed.

We appreciate the reviewer’s suggestion. In the revised manuscript, we have clarified that PRRT2-mediated regulation of Nav1.2, together with its regulation of Nav1.6, may contribute to neuronal and network excitability in the cortex. Please refer to Page 15, Line 439-440.

(2) Slow inactivation is generally considered to develop over timescales ranging from hundreds of milliseconds to seconds or longer. Therefore, the statement in Discussion (Page 13, line 381-382) that "slow inactivation develops on a timescale of tens of milliseconds to seconds" may not accurately reflect the conventional kinetic definition of slow inactivation and should be clarified.

We thank the reviewer for this comment. We have corrected the timescale description in the Discussion accordingly. Please refer to Page 13, Line 382.

(3) Page 14, line 417-430: "question about how Nav channel slow inactivation is regulated in cells that do not express PRRT2".

PRRT2 is unlikely to be the sole regulator of Nav channel slow inactivation. Other molecules and signaling pathways may regulate Nav channel and contribute to neuronal excitability. In addition, neuronal excitability can also be regulated through modulating other Nav properties, such as long-term inactivation or slow recovery from inactivation, as well as through modulating the activity of other ion channels, for example, Kv7.2 and Kv7.3 channels. Therefore, PRRT2-negative cells may utilize alternative mechanisms to fine-tune neuronal excitability. In its current form, this paragraph somewhat overstates the role of PRRT2 and would benefit from a more balanced discussion.

We appreciate the reviewer’s constructive suggestion. We agree that PRRT2 is unlikely to be the sole regulator of neuronal excitability or Nav channel slow inactivation. In the revised Discussion, we have clarified that PRRT2-dependent regulation of Nav channel slow inactivation represents one mechanism among several that fine-tune neuronal excitability. Other mechanisms, including regulation of potassium channels such as Kv7.2/Kv7.3, and other ion channel- or signaling- dependent pathways, may also contribute to excitability control in both PRRT2-positive and PRRT2-negative neurons. We have revised relevant paragraph to provide a more balanced discussion of alternative mechanisms. Please refer to Page 15, Line 430-438.

(4) Page 50, Figure 7-figure supplement 2: It would be helpful to include representative traces of the 1st and the last compound APs in panels B and C.

We appreciate the reviewer’s valuable suggestion. We have now added representative traces of the first and last compound action potentials, corresponding to the 1st and the 100th responses, respectively, to panels B and C of Figure 7-figure supplement 2. Please refer to Page 51, Figure 7-figure supplement 2.

**Recommendations for the authors:**

**Reviewer #2 (Recommendations for the authors):**

(1) Page 19, line 546: a sampling rate of 10kHz was used for recording Nav currents. Because the Nav channel isoforms examined in this manuscript (Nav1.1, Nav1.2, Nav1.4, Nav1.5, and Nav1.6) exhibit extremely rapid activation and inactivation kinetics, the temporal resolution provided by a 10 kHz sampling rate may not be optimal for detailed kinetic analysis. Although I am not requesting additional experiments, the authors may wish to choose a higher sampling rate (e.g., 50 kHz) in future Nav channel studies.

We thank the reviewer for this helpful suggestion regarding the sampling rate for Nav current recordings. We will adopt higher sampling rates for rapid kinetic analyses of sodium currents in our future work.

(2) Typo: Page 24, line 712-713: should "a Digidata (Molecular Devices, 1332A)" be "... 1322A"?

We thank the reviewer for pointing out this typo. We have corrected “Digidata 1332A” to “Digidata 1322A” in the relevant Method section of revised manuscript. Please refer to Page 25, Line 722.
